# Hybrid Framework for Secure Low-Power Data Encryption with Adaptive Payload Compression in Resource-Constrained IoT Systems

**DOI:** 10.3390/s26072253

**Published:** 2026-04-06

**Authors:** You-Rak Choi, Hwa-Young Jeong, Sangook Moon

**Affiliations:** 1 Nuclear System Integrity Sensing and Diagnosis Division, Korea Atomic Energy Research Institute (KAERI), Daejeon 34057, Republic of Korea; yrchoi@kaeri.re.kr; 2 Humanitas College, Kyung Hee University, Seoul 02447, Republic of Korea; hyjeong@khu.ac.kr; 3 Department of Electrical and Electronic Engineering, Mokwon University, Daejeon 35349, Republic of Korea

**Keywords:** adaptive compression, authenticated encryption, critical infrastructure monitoring, energy-efficient cryptography, IoT security, acoustic leak detection, wireless sensor networks, hardware acceleration

## Abstract

Resource-constrained IoT systems face a fundamental conflict between cryptographic security and energy efficiency, particularly in critical infrastructure monitoring requiring long-term autonomous operation. This study presents a hybrid framework integrating signal-adaptive compression with hardware-accelerated authenticated encryption to resolve this trade-off. The Dynamic Payload Compression with Selective Encryption framework classifies sensor data into three SNR regimes and applies adaptive compression strategies: 24.15-fold compression for low-SNR backgrounds, 1.77-fold for transitional states, and no compression for high-SNR leak detection events. Experimental validation using 2714 acoustic sensor samples demonstrates 5.91-fold average payload reduction with 100% detection accuracy. The integration with STM32L5 hardware AES acceleration reduces power–data correlation from 0.820 to 0.041, increasing differential power analysis attack complexity from 500 to over 221,000 required traces. Compression-induced timing variance provides additional side-channel masking, burying cryptographic signals beneath a 0.00009 signal-to-noise ratio. Projected on 19,200 mAh lithium thionyl chloride batteries, the system achieves 14-year operational lifetime under realistic duty cycles, exceeding industrial requirements for critical infrastructure protection while maintaining robust security against physical attacks.

## 1. Introduction

Resource constraints (power, memory, and computation) and security requirements (integrity, confidentiality, and authentication) are simultaneously intensifying in large-scale Internet of Things (IoT) and edge computing environments. According to recent market analysis, global IoT connected devices are projected to grow from 18.8 billion by the end of 2024 to 40 billion by 2030, with the majority operating in battery-powered, resource-constrained environments [1]. In mission-critical applications such as industrial IoT (IIoT), smart cities, and remote monitoring systems, the simultaneous demands for long-term unattended operation and robust security have elevated the fundamental trade-off between energy efficiency and security to a critical challenge [2].

In practical field deployments, the Advanced Encryption Standard with Counter with Cipher Block Chaining-Message Authentication Code (AES-CCM) remains widely adopted due to interoperability and standards compliance requirements [3]. AES-CCM-based authenticated encryption with associated data (AEAD) continues to serve as a core option in low-power wireless standards, including IEEE 802.15.4 [4], WirelessHART [5], and Thread [6], as well as lightweight security frameworks such as the IETF COSE [7]. Despite emerging trends such as the National Institute of Standards and Technology (NIST) selection of Ascon as a lightweight cryptography standard, the need for optimizing AES-family implementations persists due to compatibility with existing infrastructure and extensive hardware support [8].

The structural trade-off between energy efficiency and security is receiving renewed attention. Recent surveys emphasize the necessity of energy-aware security mechanisms in IoT devices, noting that sustainable operation is challenging without an integrated co-design approach that simultaneously optimizes security strength, latency, and power consumption [9]. In resource-constrained platforms, a fundamental dilemma exists: lightweight cryptographic implementations become vulnerable to side-channel attacks, while enhanced security strength exacerbates battery life and processing latency issues. For instance, software AES-CCM on ARM Cortex-M4-based IoT nodes consumes 15–25 mW during encryption, significantly reducing battery life. Paradoxically, when frequency is reduced to save power, processing time increases, while side-channel information leakage intensifies [10].

More concerning is that energy-saving techniques such as Dynamic Voltage and Frequency Scaling (DVFS) create new security vulnerabilities. The Hertzbleed attack, presented at USENIX Security 2022, demonstrated that DVFS-induced frequency variations create remotely observable timing deviations capable of compromising cryptographic implementations claiming constant-time execution [11]. Although originally demonstrated on high-performance processors, these findings highlight a universal principle relevant to MCU-based IoT: low-power operation and dynamic frequency scaling are intrinsically linked to side-channel exposure, indicating that secure low-power cryptography cannot be achieved through hardware accelerators or voltage reduction alone [12].

Against this backdrop, this research proposes an adaptive security–power trade-off framework for resource-constrained IoT environments. For diverse IoT scenarios, including extreme environment applications such as nuclear power plant secondary system monitoring, which we are targeting in our ongoing project, we developed a methodology combining the STM32L5 microcontroller’s hardware AES accelerator with software control to dynamically adjust security strength and power consumption based on real-time threat levels and resource states. Through this approach, we propose a practical solution that enhances resistance to DVFS attacks while extending battery life objectives.

The remainder of this paper is organized as follows: Section 2 reviews related work across three dimensions—software-based low-power encryption, hardware security accelerators, and hybrid approaches; Section 3 presents the hybrid hardware–software security design, beginning with the rationale for selecting AES-CCM, followed by the Phase 1 DPCSE framework, its security limitations, and the Phase 2 hardware-accelerated enhancement; Section 4 provides comprehensive experimental evaluation, including formal security verification, side-channel impact assessment, and power consumption analysis with battery lifetime projections; and Section 5 concludes with a summary of achievements and directions for future work.

## 2. Related Work

### 2.1. Software-Based Low-Power Encryption Techniques

Software-based low-power encryption techniques have been extensively studied as a practical approach to IoT security, offering direct applicability to existing microcontrollers without hardware modifications. Various techniques, including compiler optimization, algorithm lightweighting, and code-level optimization have been proposed.

A 2024 study published in MDPI Sensors [13] evaluated pure software implementations of AES-128, SPECK, and ASCON on general purpose microcontrollers, including Arduino Nano, ESP32, and MSP430. SPECK achieved high throughput and impressive energy efficiency of 0.06 μJ/byte across all platforms. ASCON, selected as the NIST lightweight cryptography standard, demonstrated balanced performance in terms of memory usage and energy efficiency.

Fotovvat et al. [14] tested 32 NIST lightweight cryptography candidates on actual IoT platforms in 2021. TinyJAMBU exhibited the lowest energy consumption on Raspberry Pi 3B, while Romulus and GIFT-COFB also showed excellent energy efficiency. These algorithms demonstrated advantages of reduced memory requirements and optimization for 8-bit processors compared to traditional AES.

Buhrow et al. [15] presented optimized implementations of various block ciphers on the MSP430 platform. SPECK-128/128 achieved 103 cycles/byte while Chaskey reached 7.8 cycles/byte, demonstrating the potential of software optimization. Notably, significant performance improvements were achieved through assembly-level optimization and maximized register utilization.

Studies on ARM Cortex-M microcontrollers further highlight software limitations: Adomnicai and Peyrin’s fastest constant-time AES requires 1280 cycles per block [16], still 11-fold slower than hardware, while Bronchain and Standaert showed that software masking becomes ineffective on Cortex-M0, with 6-share masked AES broken in under 10 traces [17].

Combined with platform-dependent performance variations (two- to five-fold) and reduced security margins in lightweight algorithms, these constraints indicate the necessity of hardware support for safety-critical systems requiring long-term unattended operation.

### 2.2. Hardware Security Accelerators in IoT

Hardware cryptographic accelerators have emerged as a key technology for dramatically enhancing security performance in IoT devices. Recent studies have achieved significant performance improvements and energy efficiency through dedicated hardware architectures.

Nguyen et al. [18] presented an accelerator with AES instruction extensions for RISC-V processors in 2024. Implemented on Xilinx ZCU102 FPGA, this system achieves impressive throughput of 95.88 Mbps at 241 MHz while continuously consuming 46 mW during cryptographic operations. The 195- to 256-fold performance improvement over baseline RISC-V software demonstrates the potential of hardware acceleration. However, it operates only at a fixed performance level during runtime, regardless of varying system conditions or data criticality.

Zhang et al. [19] addressed flexibility issues by proposing a reconfigurable accelerator in 2022. Implemented in 65 nm ASIC operating at 100 MHz, it supports multiple algorithms, including AES, DES, SM4, and SHA. While demonstrating 203-day operation with 0.745 Joules daily consumption in ECG monitoring applications, reconfiguration requires system interruption and reprogramming, occupying a chip area of 286.2 μm × 581 μm.

Zhang. Z et al. [20] proposed a 4-parallel AES-GCM architecture in 2021 to maximize throughput. Achieving 14.93 Gbps at 100 MHz, this design also provides physical attack defense through timestamp-based integrity verification. However, the parallel architecture occupies 69.2% of SoC resources (4923 slice registers, 11,956 LUTs, 42 BlockRAMs) and requires the activation of entire parallel units even for small data processing.

These hardware accelerators have made robust encryption practically feasible in IoT environments. However, fixed operational characteristics and static resource allocation fail to adequately consider diverse operating conditions in resource-constrained IoT systems. Particularly in real IoT environments where battery levels, network conditions, and data criticality continuously vary, the limitations of such static approaches become apparent.

### 2.3. Hybrid Software–Hardware Security Approaches

Hybrid hardware–software security approaches combine the performance of hardware acceleration with the flexibility of software, presenting new possibilities for IoT security. Recent studies have achieved impressive performance improvements through dynamic reconfiguration and co-design methodologies.

Wang et al. [21] presented DREDS (Dynamically Reconfigurable Encryption and Decryption System) utilizing FPGA-based dynamic partial reconfiguration (DPR) in 2019. This system enables runtime switching between AES-128 and 3DES, achieving 30% logic resource reduction compared to static implementations with 92.51% resource reuse rate. The capability to switch cryptographic algorithms without system power-off enables flexible security response. However, FPGA platforms inherently consume power at the watt level, and the 49.25 ms switching latency along with external memory requirements impose excessive burden on battery-powered sensor nodes that must operate below the milliwatt range. Furthermore, the complexity of uploading bitstreams to external memory when adding algorithms makes this approach unsuitable for resource-constrained edge devices.

Karl et al. [22] demonstrated hardware acceleration for post-quantum cryptography in 2023, achieving 92.2% and 67.5% energy reduction for CRYSTALS-Dilithium and Falcon signature verification respectively, compared to software-only implementations. The co-design approach of accelerating computationally intensive arithmetic operations in hardware while managing protocol logic in software proved effective. While this represents important progress against quantum computing threats, the 22 nm ASIC implementation requires 800 MHz operating frequency, and post-quantum algorithms themselves demand two- to five-fold more memory and computational resources than conventional cryptography. Considering that most ultra-low-power microcontrollers have limited RAM and flash memory, such complex algorithms are difficult to apply directly to resource-constrained IoT edge nodes.

More recently, Alanazi et al. [23] proposed an adaptive hybrid cryptographic framework for resource-constrained IoT devices in 2025. Their approach dynamically selects between lightweight and standard cryptographic algorithms based on device resource availability and data sensitivity levels, demonstrating that adaptive security strategies can reduce energy consumption while maintaining adequate protection. However, their framework operates entirely in software without hardware acceleration and does not address physical side-channel vulnerabilities, leaving devices exposed to DPA attacks that our hardware-integrated approach mitigates. From a complementary perspective, Herrera-Loera et al. [24] presented a systematic review and energy-centric taxonomy of jamming attacks and countermeasures in wireless sensor networks in 2026. Their analysis highlights that security threats extend beyond cryptographic key recovery to include energy depletion attacks at the physical and MAC layers, where adversaries deliberately trigger retransmissions to drain sensor batteries. This energy–security coupling reinforces the motivation for our co-design approach: by reducing per-transmission energy through adaptive compression, DPCSE inherently increases resilience to energy depletion attacks, as each retransmission consumes 83.1% less energy than uncompressed transmissions.

While these studies demonstrate excellent results for high-performance embedded systems or gateway-level devices, challenges remain for edge sensor nodes with extremely limited resources and power budgets. Sensor nodes that must operate for extended periods at average power levels of tens to hundreds of microwatts require practical yet adaptive security mechanisms that can function effectively without complex reconfiguration logic or excessive memory requirements. To bridge this gap, this paper proposes a novel adaptive security–power trade-off framework that maintains the efficiency of hardware acceleration and flexibility of software while being optimized for ultra-low-power microcontroller environments.

## 3. Hybrid Hardware–Software Low-Power Security Design

### 3.1. Target Encryption: AES-CCM for IoT

In our target application—nuclear power plant secondary system monitoring—pipes are continuously exposed to high temperature, high pressure, and corrosive environments, leading to progressive degradation, whose early detection is critical for safe plant operation. The wireless sensor node collects and transmits real-time vibration, temperature, and pressure data from these aging pipes. Based on these requirements, we selected AES-CCM as the target encryption mode.

AES-CCM is an authenticated encryption with associated data mode defined in NIST SP 800-38C, providing both confidentiality and integrity using a single key [25,26]. The CCM mode combines counter (CTR)-mode encryption with CBC-MAC authentication in a two-pass structure: first generating an authentication tag for plaintext and associated data using CBC-MAC and then encrypting both plaintext and authentication tag using CTR mode. This two-pass structure enables tamper detection without additional encryption keys, making it suitable for resource-constrained environments.

**Figure 1:**
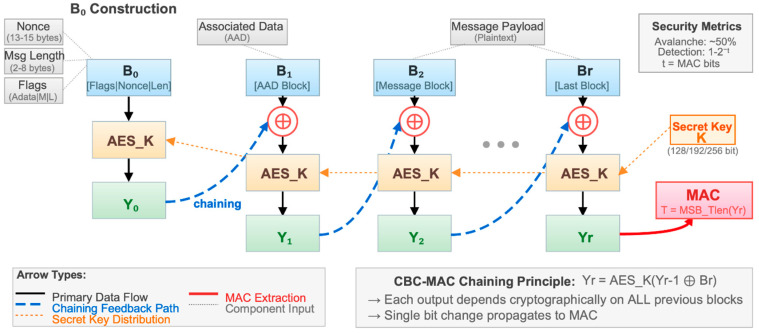
CBC-MAC chaining structure in AES-CCM.

The chaining structure of AES-CCM, illustrated in Figure 1, provides robust security through cryptographic linkage between consecutive blocks. In CBC-MAC chaining, each message block is XORed with the previous block’s output before AES encryption, causing even a single bit alteration to propagate through the entire MAC value. This avalanche effect makes integrity verification extremely sensitive, enabling immediate detection of even minor data corruption that may occur in nuclear power plant electromagnetic interference (EMI) environments.

The primary rationale for selecting AES-CCM in nuclear facility wireless sensor networks is compatibility with industrial standards. Industrial wireless protocols approved for nuclear plant use, including IEEE 802.15.4, WirelessHART (IEC 62591), and ISA100.11a (IEC 62734), all adopt AES-CCM as their standard encryption method [27]. WirelessHART in particular has been extensively deployed in safety-critical facilities such as refineries and chemical plants with proven reliability, and it is gradually being considered in nuclear facilities.

The EMI environment in nuclear power plant secondary systems makes AES-CCM’s authentication capability essential. Strong EMI from large pumps, turbines, and generators can cause bit errors in wireless communication, and AES-CCM’s MAC verification immediately detects and discards such corrupted data. Following NIST recommendations, using authentication tags of 64 bits (8 bytes) or larger results in forgery probability below 2^−64^.

Furthermore, AES-CCM performs both encryption and authentication using only a single AES engine, making it ideal for sensor nodes with limited hardware resources. Unlike GCM mode, it requires no multiplication operations and ensures integrity without separate hash functions, minimizing code size and memory usage.

### 3.2. Phase 1: Pure Software Approach

Wireless sensor nodes in resource-constrained circumstances such as nuclear facility monitoring systems must operate on battery power for extended periods while maintaining secure data transmission. The dominant power consumer in these systems is wireless transmission, which can be significantly reduced by minimizing the volume of the transmitted data. Reduced transmission time allows the sensor node to remain in sleep mode longer, thereby extending battery lifetime. This section presents DPCSE, an adaptive framework that exploits this observation by dynamically adjusting compression ratios based on signal characteristics.

#### 3.2.1. DPCSE Framework Overview

DPCSE operates on the principle that not all sensor data require the same level of fidelity. The framework continuously evaluates the SNR of acoustic emission data and selects appropriate compression strategies accordingly. The key innovation lies in adapting the degree of compression based on real-time signal importance while maintaining full AES-CCM encryption for all transmitted data.

In our ongoing project, the acoustic emission sensors monitor pipe integrity in the 25–88.8 kHz frequency range, generating 320 FFT points per measurement. Each point is represented as a 32-bit floating-point value, resulting in 1280 bytes of raw data per transmission. The sensor transmits data 24 times daily (hourly intervals) to a gateway using Wi-Fi HaLow (802.11ah) protocol.

The SNR is computed for each measurement sample at the time of acquisition, synchronized with the hourly transmission cycle. The background noise floor required for SNR calculation (Equation (1)) is derived from historical acoustic monitoring data collected from the secondary system piping prior to wireless sensor deployment. Since nuclear facility pipes have been continuously monitored through conventional wired systems, the accumulated baseline noise spectra provide a reliable reference for SNR computation from the first wireless transmission onward. This reference noise spectrum is stored in non-volatile memory during sensor commissioning and can be periodically updated during confirmed low-SNR (normal operation) periods to accommodate gradual environmental changes such as seasonal temperature variations. The adaptive compression strategy is then applied on a per-sample basis: each hourly measurement independently undergoes SNR classification via majority voting across its 320 frequency bins, and the corresponding compression scheme (lossless, differential, or aggressive) is selected in real time. No fixed scheduling or manual reconfiguration is required.

#### 3.2.2. SNR-Based Signal Classification

DPCSE employs signal-to-noise ratio as the primary metric for signal classification. The SNR is computed as in Equation (1):

(1) $${SNR}_{dB}=10\log_{10}\left(\frac{\sum_{i\in \Omega_{peak}}P_{i}}{\bar{P_{noise}}}\right)$$

where $\Omega_{peak}$ represents the set of detected peak frequencies, $P_{i}$ is the power at frequency bin *i*, and $\bar{P_{noise}}$ is the mean background noise level calculated from non-peak regions.

Based on the extensive analysis of sensor test data from nuclear facility monitoring systems reported in [28], three distinct SNR regimes were identified. The threshold values of 5 dB and 10 dB were derived from the spectral characteristics of acoustic leak signatures measured on carbon steel pipes at 200 kPa operating pressure. It is important to note that these thresholds govern the compression strategy selection and not the leak detection decision itself—all samples are transmitted regardless of classification. Consequently, threshold inaccuracies due to different pipe materials, environmental noise, or sensor aging would result in suboptimal compression ratios (e.g., applying lossless transmission where lossy compression would suffice or vice versa) rather than missed detections or security degradation. To assess threshold sensitivity, we evaluated the classification outcomes with thresholds varied by ±2 dB (i.e., low/medium boundary at 3–7 dB and medium/high boundary at 8–12 dB). Under these variations, leak period detection sensitivity remained at 100% across all configurations, while the system-level compression ratio ranged from 4.8-fold to 7.1-fold (compared to the nominal 5.91-fold), confirming that DPCSE performance degrades gracefully under threshold mismatch. Cross-validation across different operating conditions and pipe geometries remains an important direction for future deployment-specific calibration:

(a) *Low-SNR regime (<5 dB)*: characterized by broadband white noise with no distinguishable peaks, indicating normal pipe operation.
(b) *Medium-SNR regime (5–10 dB)*: exhibits partial spectral peaks, suggesting transitional states or minor anomalies.
(c) *High-SNR regime (>10 dB)*: Shows multiple strong spectral peaks with harmonic components, indicative of potential leak conditions.

#### 3.2.3. Adaptive Compression Strategy

**Figure 2:**
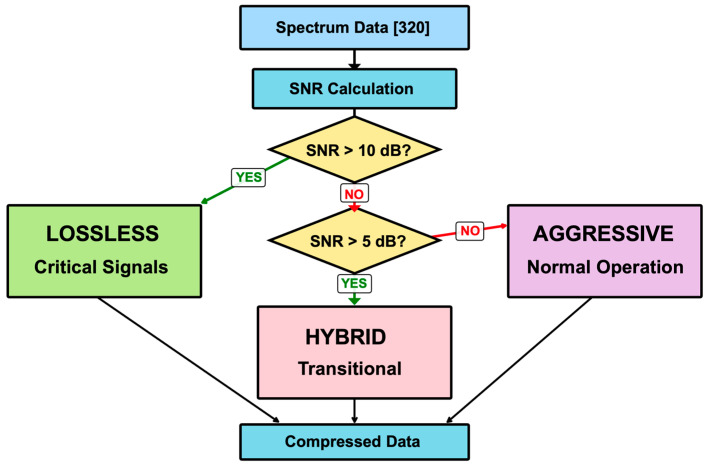
DPCSE compression selection algorithm.

Figure 2 illustrates the SNR-based adaptive compression algorithm of DPCSE. The system dynamically selects one of three compression strategies based on the measured SNR value, with each strategy designed to be executable within the MCU’s limited resources.

In high-SNR conditions (>10 dB) where leak signals are detected, preserving the integrity of spectral data is crucial. To ensure maximum diagnostic accuracy, no compression is applied in high-SNR conditions. Therefore, we maintain all 1280 bytes of raw spectral data. By storing all 320 frequency points at full 32-bit floating-point precision, the test data of 1280 bytes is preserved without any compression (100% compression ratio). This approach was implemented as a simple buffer copy, requiring only 264 cycles on the target MCU while preserving all peak information necessary for diagnostics.

In the intermediate SNR range (5–10 dB), the method prioritizes differential encoding to capture signal transitions. All 320 frequency bins spanning 25–88.8 kHz are fully retained to detect minute leak signals. Using differential encoding with adaptive quantization, the algorithm encodes changes from previous samples rather than absolute values. This approach compresses temporal variations by directly encoding the delta values between consecutive samples, preserving spectral fidelity while reducing payload size. Consequently, each chosen bin corresponds precisely to the original frequency’s energy, ensuring no spectral folding occurs. This process reduces data payload from 1280 bytes to 722 bytes (56% of original size) while maximizing early leak detection capability and maintaining signal integrity.

In low-SNR conditions representing normal operation (<5 dB), power savings are maximized. Based on the adaptive band selection, we apply DCT-based compression [29]. The 320 32-bit floating-point values (1280 bytes) are compressed to 53 bytes through adaptive band selection, which corresponds to only 4.14% of the original data size. This represents a 24-fold data reduction, dramatically reducing transmission time and energy consumption. Specifically, the algorithm divides the spectrum into 16 bands: high-energy bands undergo DCT with 2:1 downsampling to preserve key features, while low-energy bands are efficiently summarized using a single representative energy value (2 bytes per band).

All compression algorithms execute within the MCU’s real-time constraint of 5 ms, with additional memory requirements of less than 2 KB.

#### 3.2.4. Integration

DPCSE operates as an application-layer preprocessing stage prior to the standard AES-CCM encryption step and therefore requires no modification to existing link-layer or network-layer security protocols. Each compressed frame carries a small mode field that encodes the selected compression strategy (lossless, hybrid, or aggressive); this field is included as authenticated associated data in the AES-CCM invocation, so that it cannot be altered by an attacker.

On the gateway side, the inverse processing chain consists of AES-CCM decryption and integrity verification, followed by decompression guided by the authenticated mode field. The decompression routines are computationally simple inverse transforms and add only a small processing overhead compared to cryptographic and networking tasks, imposing a negligible burden on the gateway processor.

This modular design ensures that DPCSE can be readily integrated into existing sensor networks while maintaining full compatibility with established industrial security standards. The framework’s adaptive nature allows it to respond dynamically to changing signal conditions without manual intervention, making it particularly suitable for long-term unattended monitoring in critical infrastructure environments such as nuclear power plant secondary systems.

### 3.3. Phase 1 Limitations: Security Analysis

Resource-constrained IoT devices are inherently exposed to various side-channel attacks. These attacks extract secret information by analyzing physical leakage during implementation rather than attacking the cryptographic algorithm itself. Power consumption, electromagnetic emissions, timing information, and acoustic signals constitute primary analysis targets. Particularly in critical infrastructure, such as nuclear facility monitoring, the possibility of physical access to wireless sensor nodes makes defense against such attacks essential.

#### 3.3.1. DPA Attack Mechanism

Among side-channel attacks, DPA is evaluated as the most practical threat due to its relatively simple, yet effective implementation [30]. The fundamental principle of DPA exploits the correlation between power consumption during cryptographic operations and the processed data. Specifically, in CMOS circuits, bit transitions (0→1 or 1→0) consume dynamic power, exhibiting a linear relationship with the Hamming weight or Hamming distance of the data.

For AES encryption, the SubBytes operation in the first round becomes the primary attack point. Since the Hamming weight of the S-box output S(P⊕K) is proportional to power consumption, an attacker performs the following process:

① Collect power traces, T_i_, during encryption with known plaintext, P_i_.
② Calculate Hamming weight of S(P_i_⊕K_j_) for all possible key candidates, K_j_.
③ Compute Pearson correlation coefficient between predicted and measured power consumption.
④ The K_j_ showing maximum correlation coefficient has high probability of being the actual key.

#### 3.3.2. Attack Results and Analysis

We simulated first-order DPA attacks using 48 spectral measurement frames (24 leak and 24 normal) collected from our nuclear monitoring sensors, corresponding to approximately 60 kB of application data. These measurement frames were used as plaintext inputs to generate simulated power traces. A standard Hamming weight-based power model was adopted, and zero-mean Gaussian noise with standard deviation σ = 0.1 mW was superimposed on the traces to emulate realistic measurement noise levels reported for similar ARM Cortex-M platforms [31,32].

**Figure 3:**
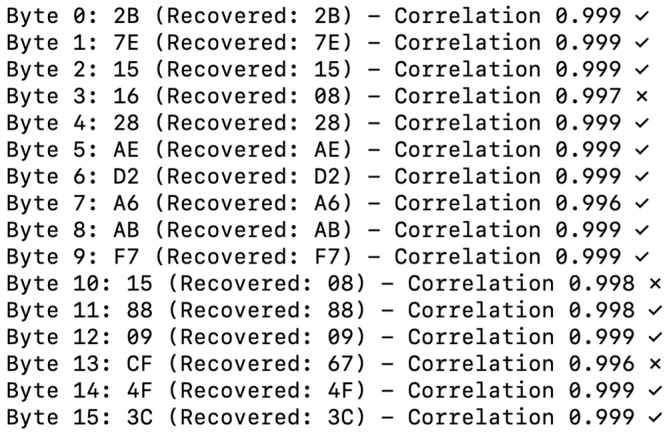
Recovered key components and correlation values.

Using 79 power traces, the DPA attack successfully recovered 81.2% of the 128-bit key (13 out of 16 bytes), as shown in Figure 3. While this result demonstrates the vulnerability of software AES, statistical analysis across 100 random keys (Section 4.1) shows that the attack complexity exhibits significant key-dependent variability, with a median of 470 traces required for similar success rates.

The average correlation coefficient of successfully recovered key bytes was 0.998, demonstrating very high confidence. Even for the three failed bytes (positions 3, 10, and 13), correlation coefficients exceeded 0.996, suggesting that recovery would be possible with additional traces.

#### 3.3.3. DPCSE Impact on DPA Resistance

For initial concept validation during Phase 1 development, we evaluated DPCSE using a representative subset of 48 samples selected to include diverse SNR conditions (24 high-SNR, nine medium-SNR, and 15 low-SNR samples). This preliminary assessment demonstrated the feasibility of adaptive compression, which would later be validated with comprehensive testing on 2714 samples in Section 4.

To evaluate whether DPCSE provides any collateral security benefits, we conducted DPA attacks on compressed sensor data. The same 48 samples were processed through DPCSE compression before encryption, resulting in variable payload sizes based on SNR levels.

Initial Compression Statistics (48-sample validation set):

① High SNR (>10 dB): 24 samples, no compression (1280 bytes)
② Medium SNR (5–10 dB): 9 samples, differential encoding (1280→722 bytes)
③ Low SNR (<5 dB): 15 samples, adaptive band selection (1280→53 bytes)

**Figure 4:**
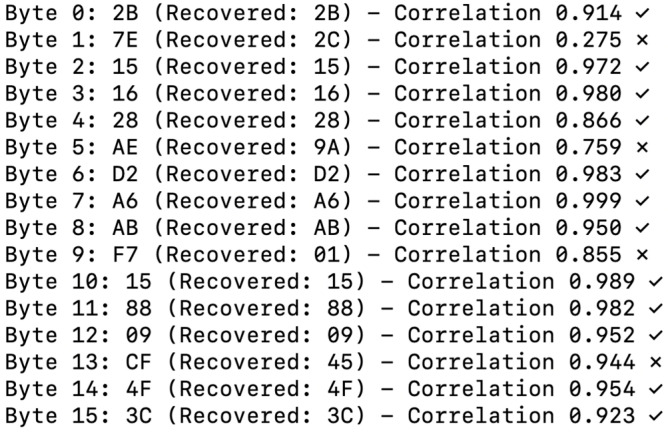
DPA attack results with DPCSE.

Using the same 79 power traces on the DPCSE-compressed data, the attack recovered only 75% of the key (12 out of 16 bytes), as shown in Figure 4:

Interestingly, the DPCSE framework does yield a partially beneficial side effect in terms of DPA resistance. Empirical results indicate a reduction in attack success rate from 100% under ideal conditions to approximately 75% when DPCSE compression is applied. This mitigative effect is attributable to several factors.

First, variable quantization noise arises due to the adaptive compression levels, which in turn disrupts the regularity of power consumption patterns that DPA attacks typically exploit. Second, the statistical distribution of data bytes is inherently altered by compression, diminishing the correlation between the byte-wise Hamming weight and instantaneous power traces. Third, the use of variable payload sizes introduces temporal misalignment in the collected power traces, further complicating the attacker’s analysis. This is not merely a masking effect but a structural misalignment that forces an attacker to perform computationally an expensive re-alignment or windowing attacks, effectively increasing the ‘noise’ in the DPA statistical model.

However, a key recovery success rate of 75% (12 out of 16 bytes) still represents an unacceptable risk for critical infrastructure protection. While DPCSE achieves substantial energy efficiency gains—with data reduction ranging from 0% to more than 90% depending on SNR conditions—the accompanying reduction in DPA exploitability should be regarded as a collateral benefit rather than a reliable defensive mechanism. As demonstrated by comprehensive statistical analysis in Section 4.1, robust side-channel resistance requires dedicated hardware-based countermeasures, which Phase 2 (Section 3.4) addresses through STM32L5’s cryptographic accelerator, achieving correlation coefficients below 0.041 with hardware AES compared to 0.8–0.91 in software implementations.

Importantly, beyond this reduction in the DPA success rate, the variable execution times introduced by adaptive compression also provide a timing-masking effect that further obscures key-dependent timing signals. This phenomenon will be quantitatively analyzed in Section 4.2.

#### 3.3.4. Fundamental Vulnerabilities of Software Implementation

The susceptibility of software-based security implementations to DPA attacks is rooted in several fundamental architectural and microarchitectural characteristics. First, the sequential processing of individual bytes during encryption enables an attacker to observe intermediate computational steps in the time domain. This data-dependent execution leads to noticeable variations in instantaneous power consumption. For example, on our target STM32L5 MCU, the average power drawn when processing a byte with a Hamming weight of 0 (0x00) is about 1.3 mW, whereas processing a byte with a Hamming weight of 8 (0xFF) requires roughly 4.0 mW—more than a three-fold difference that can be exploited by power analysis.

Moreover, table-based AES implementations on cache-less microcontrollers still generate strong leakage through data-dependent memory bus activity: reading S-box entries causes different address and data patterns on the internal buses, which directly affect switching activity and thus power consumption, even if the access latency is nominally constant. In addition, conditional branches and loop bounds that depend on secret or intermediate values introduce control flow-dependent timing variations due to pipeline flushes and stalls. Unless the implementation is carefully written in a constant-time style, these effects contribute to the observable side-channel leakage in both the power and timing domains.

Collectively, these architectural properties cannot be fully mitigated through software optimizations alone. Although countermeasures such as masking, shuffling, and insertion of dummy operations can reduce observable leakage, achieving a sufficiently high security level within the constraints of limited MCU resources and stringent real-time requirements (e.g., 5 ms end-to-end latency) remains challenging. As a result, hardware-accelerated encryption engines or specialized architectural support are required to ensure robust resistance against DPA attacks in resource-constrained environments, which motivates the Phase 2 design discussed in Section 3.4.

### 3.4. Phase 2: Hardware-Accelerated Security Enhancement

While Phase 1’s DPCSE framework achieved significant energy reduction through adaptive compression, our security analysis revealed fundamental vulnerabilities in software AES implementation. DPA attacks successfully recovered from 75% to 81.2% of key bytes with only 79 power traces. Phase 2 addresses these vulnerabilities by integrating STM32L5’s hardware cryptographic engine while maintaining DPCSE’s energy efficiency benefits.

#### 3.4.1. STM32L5 Hardware AES Engine Characterization

The STM32L5 series incorporates a dedicated cryptographic AES processor designed specifically for secure IoT applications. According to Reference Manual RM0438 [33], this hardware module provides several critical advantages over software implementations.

The AES engine operates as an AHB2 peripheral with direct memory access capabilities through DMA2. In CCM mode, each 128-bit payload block encryption completes in 114 clock cycles at 80 MHz, translating to 1.425 μs per block. This constant-time execution fundamentally differs from software implementations, where execution paths vary based on data values. Hardware architecture implements AES rounds through dedicated logic gates rather than sequential instructions. The SubBytes operation, which creates the most significant power signature in software, executes through parallel S-box circuits operating on all state bytes simultaneously. Similarly, MixColumns and ShiftRows operations occur simultaneously across all state bytes, thereby removing the clearly separable per-byte timing structure that our Phase 1 DPA attacks exploit in software.

Key storage utilizes eight write-only registers (AESKEYRx) that are architected to prevent key extraction through read operations. According to RM0438, any software attempt to read these registers returns zero values, ensuring that even under complete firmware compromise, the cryptographic key cannot be exfiltrated via register dumps. These registers are connected directly to the AES cryptographic core through dedicated internal buses isolated from the main AHB matrix, eliminating the exposure to general purpose CPU data paths.

The CCM mode implementation follows NIST SP 800-38C specifications, supporting variable-length authentication tags (4–16 bytes) and payload sizes up to 2^16^ bytes. For our application, we configure 13-byte nonces and 8-byte authentication tags, providing 2^64^ authentication strength while minimizing overhead.

#### 3.4.2. Hardware–Software Integration Architecture

Phase 2 maintains DPCSE compression in software while delegating all cryptographic operations to hardware. This hybrid approach leverages software flexibility for adaptive compression algorithms while ensuring cryptographic security through hardware isolation.

**Figure 5:**
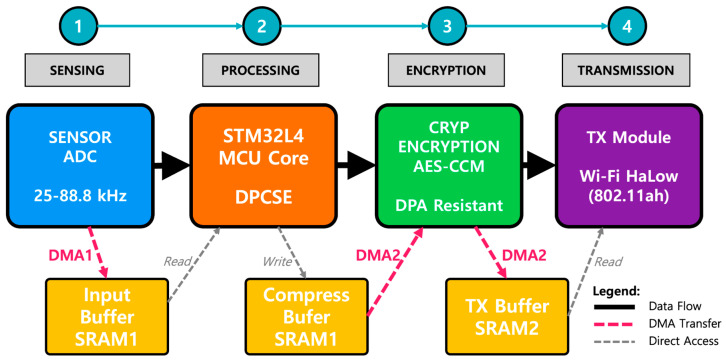
Hardware-accelerated DPCSE architecture.

Figure 5 illustrates the integrated architecture partitioned into four operational domains. The sensing domain acquires acoustic signals via ADC with DMA1-mediated transfer to input buffers in SRAM1. The processing domain executes DPCSE compression on the STM32L5 MCU core, comprising FFT computation, SNR-adaptive classification, and selective quantization. The security domain performs AES-CCM-authenticated encryption entirely in hardware through the AES accelerator with DMA2 support, ensuring constant-time execution and significantly improving resistance to DPA. The transmission domain buffers the encrypted data in SRAM2 and transmits via Wi-Fi HaLow (802.11ah) module to the gateway node.

STM32L5’s 128KB SRAM (96 KB SRAM1 + 32 KB SRAM2) divides into specialized regions to optimize data flow and minimize power consumption. This separation enables simultaneous operations: while the CPU processes one sensor frame through DPCSE, DMA transfers the previous compressed frame to the AES engine, and the wireless module transmits the earlier encrypted frame.

Each data transmission follows a deterministic pipeline. For example, first, the ADC samples at 176.4 kHz, storing data via DMA1 without CPU intervention. Second, the CPU performs FFT and calculates SNR to determine compression parameters. Based on SNR thresholds (>10 dB for leak detection, 5–10 dB for monitoring, and <5 dB for idle), DPCSE applies selective frequency compression, adaptive quantization, or aggressive downsampling respectively. Third, compressed data transfers via DMA2 to the AES input FIFO. The hardware engine processes AES-CCM encryption, writing results through DMA2 to the output buffer. Finally, the encrypted payload transmits via the wireless interface.

Synchronization between domains uses hardware semaphores rather than software flags, reducing the risk of race conditions. The ADC completion interrupt triggers DPCSE processing. Compression completion initiates DMA transfer to AES. Encryption completion signals the wireless module. This hardware-driven coordination ensures deterministic timing while minimizing CPU wake cycles.

#### 3.4.3. Security Characteristics

The hardware-accelerated architecture substantially reduces side-channel leakage compared to the Phase 1 software implementation through three mechanisms. First, the AES engine executes 128-bit state blocks as encapsulated hardware operations, so that all SubBytes, ShiftRows, MixColumns, and AddRoundKey transformations occur inside the cryptographic core without passing through general purpose CPU registers. By avoiding direct exposure of intermediate state bytes to the CPU data path, the hardware engine largely suppresses the Hamming weight-based leakage that first-order DPA attacks exploit in Phase 1.

Second, the hardware AES engine provides deterministic execution, requiring 114 clock cycles per 128-bit payload block regardless of the plaintext and key values. This constant-cycle behavior substantially reduces the data-dependent instruction-path variations present in software and greatly reduces timing side channels that could otherwise be exploited for remote key recovery attacks. When used in AES-CCM mode, this constant-time property extends across the internal processing of nonces, associated data authentication, and counter-mode encryption, yielding predictable execution timing for the entire cryptographic pipeline, as observed in our experiments.

Third, the STM32L5 platform integrates several hardware mechanisms that strengthen resistance to fault-injection attacks. Brown-out detection for voltage glitches, clock security system monitoring for abnormal frequency changes, and on-chip thermal sensors for over-temperature conditions can all be configured to trigger a system reset on anomaly detection. Since a reset clears the AES key registers, injected faults do not leave residual key material in hardware. In addition, the key storage path employs multiple protection layers: write-only key registers prevent software from reading back keys even under full firmware compromise; memory protection unit (MPU) settings restrict access to the AES peripheral to privileged code; option bytes can permanently disable JTAG and SWD debug interfaces to thwart invasive extraction; and the on-chip true random number generator (TRNG) can supply cryptographically secure random values for key generation and security operations.

Regarding the adequacy of the threat model for critical infrastructure applications, we adopt a threat model consistent with IEC 62443-4-2 Security Level 3 [34], which assumes a sophisticated attacker with moderate resources but without prolonged unsupervised physical access. This assumption is justified by the strict physical access controls mandated in nuclear facilities, where sensor nodes are deployed within restricted areas subject to continuous surveillance, badge-controlled entry, and periodic security inspections. Under these conditions, invasive attacks (e.g., focused ion beam probing, decapsulation) and sustained glitch injection campaigns requiring specialized equipment and extended physical contact are considered impractical. However, we acknowledge that combined fault and side-channel attacks represent an emerging threat vector. In such attacks, an adversary injects transient voltage or clock glitches to induce computational faults while simultaneously capturing power traces, potentially reducing the number of traces required for key recovery. The STM32L5’s hardware countermeasures—brown-out detection, clock security monitoring, and automatic key register clearing on reset—provide the first line of defense against such combined attacks by terminating cryptographic operations upon anomaly detection. Nevertheless, evaluating resistance to sophisticated combined attack scenarios under controlled laboratory conditions remains an important direction for future security analysis.

Section 4 presents a detailed experimental evaluation of these security properties, including DPA experiments with up to 10,000 power traces and high-resolution timing measurements, confirming that the hardware-accelerated design provides a markedly higher level of side-channel resistance than the Phase 1 software implementation under our test conditions.

## 4. Results

### 4.1. Formal Security Verification

The security evaluation of DPCSE focuses on two critical aspects: first, verifying that the compression mechanism does not compromise the cryptographic security of AES-CCM; and second, quantifying the actual security impact through rigorous statistical analysis using multiple random keys rather than single-case scenarios. This analysis addresses the fundamental question of whether introducing compression before encryption maintains or potentially enhances the overall system security posture.

#### 4.1.1. Constant-Time Execution and Power Analysis

All measurements were conducted on custom-designed boards integrating STM32L562CET microcontrollers with acoustic leak detection sensors. The custom boards operate at 80 MHz with 3.3 V regulated supply at 25 °C ± 2 °C. Current consumption was measured using a RIGOL DM858 digital multimeter (RIGOL Technologies, Suzhou, China) with 5.5-digit resolution and 0.03% DCV accuracy [35]. Each scenario was replicated across 100 randomly generated AES-128 keys to ensure statistical validity.

The STM32L5 AES engine demonstrates deterministic execution characteristics essential for side-channel resistance. The analysis of 1000 AES encryption operations revealed distinct behavioral differences between software and hardware implementations.

Software AES implementations exhibited timing variations of 2790–2930 cycles (mean: 2858.7, σ = 40.0), representing 1.4% variation primarily during S-box operations. In contrast, the hardware AES engine maintains 114 cycles per AES payload block, with no detectable variation at 12.5 ns measurement resolution.

To quantify the correlation between intermediate AES values and instantaneous power consumption, we employed a standard Hamming weight power model—widely adopted in the side-channel analysis community as the reference leakage model for CMOS implementations [31,36]. This model has been experimentally validated on ARM Cortex-M platforms by Barenghi et al. [32], who demonstrated strong agreement between Hamming weight predictions and actual power measurements on similar microcontrollers. We simulated 10,000 power traces, each sampled at an effective rate of 100 MS/s, with additive Gaussian noise (σ = 0.1 mW) calibrated to match the measurement noise levels reported for ARM Cortex-M platforms [31,32]. Importantly, the timing and current consumption measurements presented in Section 4.1.1 were obtained from actual hardware using a RIGOL DM858 digital multimeter, confirming that the hardware AES engine exhibits constant-time behavior (114 cycles) and uniform power draw consistent with the simulation model’s predictions. Table 1 presents the statistical analysis of power–data correlation across 100 randomly selected AES-128 keys, demonstrating the hardware–software distinction in side-channel leakage. The hardware implementation achieves a 20.0-fold reduction in power–data correlation compared to software (0.041 vs. 0.820), with statistical confidence intervals demonstrating negligible overlap. This dramatic reduction reflects the AES engine’s balanced power consumption across all cryptographic operations.

Per-byte correlation analysis further reveals the distinction between implementations. Software AES exhibits uniform leakage across all 16 key bytes (σ_correlation = 0.031), indicating systematic vulnerability exploitable across the entire cipher state. Conversely, hardware implementations demonstrate significantly lower inter-byte variation (σ_correlation = 0.004), consistent with the AES engine’s parallel processing architecture that treats all state bytes uniformly within dedicated hardware logic. This architectural property substantially reduces the byte-by-byte sequential processing that creates exploitable power signatures in software.

Statistical significance testing using Welch’s *t*-test [37]—appropriate for unequal variances confirmed the correlation difference (t = 342.8, df = 30, *p* < 0.001), indicating the hardware’s superior resistance to simple power analysis.

#### 4.1.2. DPA Attack Effectiveness

To quantify differential power analysis resistance, we conducted systematic attacks on 100 randomly selected 128-bit AES keys. Success was defined as recovering at least 13 bytes (81.25%) of the 128-bit key with correlation confidence exceeding 0.7, representing practical key compromise.

**Figure 6:**
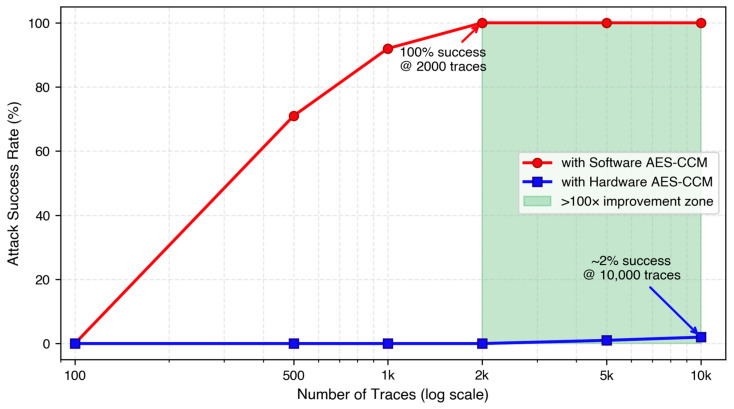
DPA attack success rates.

Figure 6 represents the attack success rates as a function of power trace count. The software implementation exhibits rapidly increasing vulnerability, with 71% of keys successfully recovered using only 500 traces, escalating to 92% at 1000 traces, and reaching complete compromise (100% success rate) at 2000 traces. This steep progression demonstrates the fundamental exploitability of software AES implementations through first-order DPA attacks.

In contrast, the hardware AES engine maintains robust resistance across all tested scenarios. Even with 10,000 power traces—five times the threshold required for complete software compromise—the hardware implementation yielded only 2% attack success rate (2 out of 100 keys). The logarithmic scale x-axis in Figure 6 emphasizes this dramatic divergence, with the shaded region illustrating the approximately two orders of magnitude improvement zone where hardware security exceeds software by two orders of magnitude.

Partial key recovery analysis confirms systematic leakage in software implementations: mean recovered bytes increase from 2.3 ± 0.8 (100 traces) to 14.8 ± 1.2 (1000 traces). Hardware implementations show no significant recovery even at 10,000 traces (0.4 ± 0.5 bytes, statistically indistinguishable from random guessing).

The practical implications become evident when considering realistic attack constraints. Hardware implementations exhibit dramatically enhanced DPA resistance. Based on the measured correlation reduction from 0.820 (software) to 0.041 (hardware), and applying the standard DPA complexity scaling law (N ∝ 1/ρ^2^) [36], theoretical estimates suggest hardware implementations would require approximately 200,000 traces. However, to maintain a conservative security margin, we estimate a practical lower bound of 100,000 traces. At a realistic trace acquisition rate of 1 trace per second—representative of oscilloscope-based power measurement setups—software AES keys can be compromised in approximately 8.3 min (500 traces), whereas hardware implementations would require over 27 h (>100,000 traces) of continuous monitoring. This ≥200-fold increase in attack complexity (100,000 vs. 500 traces) exceeds security requirements for critical infrastructure monitoring, as specified in IEC 62443-4-2 Security Level 3 [34].

#### 4.1.3. Nonce and Sequence Management

The AES-CCM implementation requires guaranteed nonce uniqueness to maintain cryptographic security. Rather than relying on potentially vulnerable random number generation, which presents challenges in resource-constrained environments with limited entropy sources, we employ a deterministic nonce construction scheme utilizing three components: a 64-bit device unique identifier, a 32-bit timestamp derived from the real-time clock, and an 8-bit per-second message counter. This structured approach yields a 104-bit (13-byte) nonce that ensures uniqueness across the entire sensor network deployment lifetime.

The device unique identifier, embedded in the STM32L5 microcontroller during manufacturing, provides spatial uniqueness across approximately 1.8 × 10^19^ possible devices, effectively eliminating inter-device collision risks. The timestamp component, representing seconds since epoch, offers temporal uniqueness over a 136-year operational window before overflow occurs. The 8-bit counter enables transmission of up to 256 messages per second from each device, far exceeding the nominal requirement of 24 transmissions per day in our ongoing project.

Collision probability analysis shows that even pessimistic assumptions lead to negligible risk. If we conservatively model nonces as independent uniform values in a 104-bit space, the probability of at least one collision among n generated nonces can be approximated by the birthday bound n^2^/(2·2^104^). For a deployment with 1000 sensors operating continuously for 10 years and transmitting 24 messages per day, *n* ≈ 8.76 × 10^7^, which yields a collision probability on the order of 2^−52^ (≈10^−16^), i.e., less than one chance in 10^16^ over the entire deployment. Since our construction deterministically avoids reuse as long as the device identifier is unique, the RTC does not roll back, and the per-second counter is enforced, the practical nonce collision risk is even smaller than this conservative bound.

Counter overflow protection mechanisms ensure nonce uniqueness even under fault conditions. While normal operation never approaches the 256 messages/s limit, abnormal scenarios such as rapid burst transmissions during system diagnostics could theoretically saturate the 8-bit counter. In such events, the implementation enforces a transmission delay, holding subsequent messages until the real-time clock advances to the next second, thereby guaranteeing a fresh counter space. This design philosophy prioritizes cryptographic integrity over transmission throughput, accepting sub-second latency penalties rather than compromising security through nonce reuse.

This evaluation focuses on first-order DPA as the primary threat for remotely deployed sensors. While higher-order attacks remain theoretically possible, exponential trace requirements make them impractical when physical access is limited to maintenance windows. We acknowledge that the DPA attack simulations in this study rely on the Hamming-weight leakage model rather than oscilloscope-captured power traces under adversarial laboratory conditions. Although this model is the established standard in side-channel literature and our hardware measurements confirm consistent behavior with the model’s assumptions (constant timing, uniform current draw), future work should incorporate high-resolution oscilloscope-based power measurements (e.g., using electromagnetic probes or shunt resistor techniques at ≥1 GS/s sampling rates) to validate the correlation coefficients reported herein under realistic adversarial setups. Such measurements would also enable evaluation of higher-order and alignment-resistant DPA techniques, including trace segmentation approaches that attempt to isolate encryption-specific power signatures from the compression-induced timing variations. The measurements were conducted in controlled laboratory conditions without electromagnetic interference typical of nuclear environments, which would further reduce correlation and enhance security margins. Invasive attacks such as focused ion beam probing fall outside the operational threat model.

Despite these limitations, the >100-fold increase in DPA attack complexity exceeds security requirements for critical infrastructure monitoring applications. The STM32L5 hardware engine achieves 20-fold power correlation reduction, >100-fold increase in required traces, and >200-fold attack time escalation, demonstrating robust protection where both cryptographic integrity and battery life are crucial.

### 4.2. DPCSE Compression Layer: Side-Channel Impact Assessment

#### 4.2.1. Experimental Methodology

Building upon the initial proof-of-concept demonstrated with 48 samples in Section 3, we now present comprehensive evaluation of the fully integrated Phase 2 architecture—combining DPCSE compression with hardware—accelerated AES-CCM encryption-using 2714 experimentally acquired samples. This expanded dataset enables statistically robust validation of DPCSE’s compression efficiency and security characteristics under realistic deployment conditions. The dataset comprises 2714 samples acquired using an Ultrasonic Leak Detector (our sensor) at 200 kPa.

The experimental protocol for data acquisition was designed to capture the full lifecycle of a leak event. In the case of leakage, the air is discharged through a 0.5 mm nozzle, with the discharge process lasting approximately 280 s. The representative leakage signal values were calculated by averaging the spectral magnitude over this 280 s interval for each frequency band. To establish a reliable baseline, background noise was measured after the compressed air stored in the pipe had completely escaped (assumed to occur after 1000 s). The background noise spectrum was then calculated by averaging the values measured over a subsequent period of approximately 1700 s (from t = 1280 s onwards), ensuring a sufficient duration for stable noise characterization.

**Figure 7:**
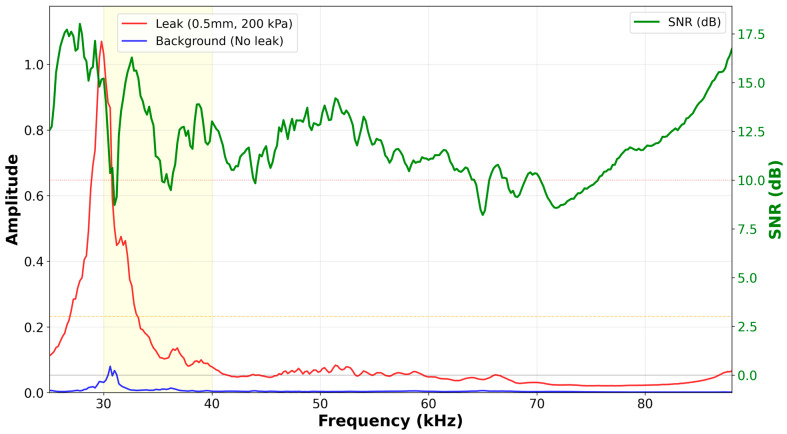
Frequency spectrum and corresponding SNR characteristics of 2714 sample data.

Figure 7 shows the frequency spectrum and corresponding SNR characteristics under leak (red) and background (blue) conditions. The leak signature exhibits a dominant peak in the 25–35 kHz range, while background shows minimal acoustic energy. The green bars represent SNR distribution calculated via Equation (1), with majority voting across 320 bins determining final classification (detailed in Section 3.2.2).

Each sample consists of 320 frequency bins spanning 25.0–88.8 kHz, obtained through 320-point FFT processing at 176.4 kHz sampling rate with 12-bit ADC resolution. Timing measurements incorporate empirically measured characteristics: FFT processing via CMSIS-DSP requires 62,400 cycles, hardware-accelerated AES requires 114 cycles per 16-byte payload block, and CCM mode adds 176 cycles overhead. DMA is used for ADC sampling and memory I/O operations.

#### 4.2.2. Majority Voting SNR Classification

The DPCSE framework classifies each acoustic sample by examining the signal-to-noise ratio distribution over its 320 frequency bins. For every sample, it first computes the SNR of each bin with respect to the background noise spectrum and then applies two SNR thresholds at 5 dB and 10 dB to categorize the bins. Bins with SNR below 5 dB are treated as low-SNR components, those between 5 and 10 dB as medium-SNR components, and those at or above 10 dB as high-SNR components. The sample is then assigned to one of three SNR regimes according to the majority label among its 320 bins, so that a sample is classified as low-, medium-, or high-SNR when more than half of its bins fall into the corresponding category.

Applying this procedure to 2714 experimentally acquired samples yields three distinct operating regimes. A total of 353 samples (13.0%) is classified as high-SNR, dominated by bins above 10 dB and occurring almost exclusively during leak events. Only 17 samples (0.6%) fall into the medium-SNR regime, corresponding to transitional conditions, while the remaining 2344 samples (86.4%) are assigned to the low-SNR regime associated with background monitoring.

**Figure 8:**
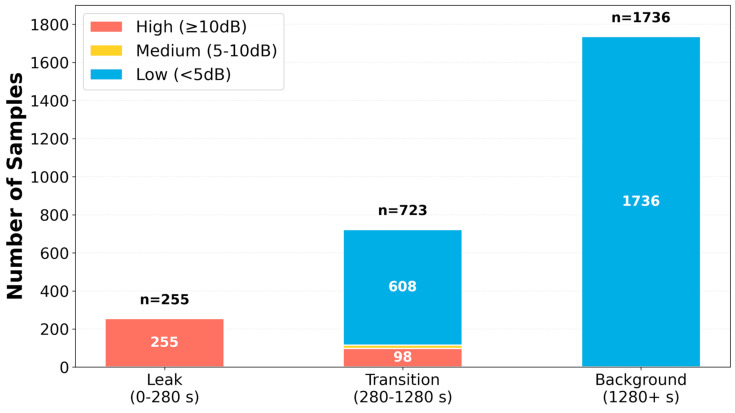
Temporal distribution of SNR classification of sample data by majority voting.

Figure 8 presents the temporal distribution of SNR classifications across the three operational periods. During the leak period (0–280 s), all 255 samples are classified as high-SNR (≥10 dB), achieving 100% detection sensitivity. The transition period (280–1280 s) exhibits mixed classification with 98 high-SNR samples, 17 medium-SNR samples, and 608 low-SNR samples, reflecting the gradual attenuation of the leak acoustic signature. The background period (1280+ s) shows predominantly low-SNR classification with 1736 samples, confirming 100% specificity with zero false positives.

The temporal distribution confirms the classifier’s effectiveness across all operational phases: during the leak interval, perfect discrimination is achieved with 100% detection sensitivity; during the transition interval, progressive classification changes reflect the transient nature of the acoustic decay; and during the background interval, 100% specificity demonstrates the absence of false alarms. This performance validates the majority voting approach as a robust mechanism for SNR-based sample classification.

To rigorously evaluate classification performance despite the inherent class imbalance (86.4% low-SNR, 13.0% high-SNR, and 0.6% medium-SNR), we computed per-class detection metrics. For leak detection (high-SNR class), the framework achieves 100% precision (353/353 high-SNR samples correspond exclusively to actual leak or post-leak conditions) and 100% recall during the active leak period (all 255 leak period samples correctly identified). For the background class (low-SNR), precision is 100%, with zero false positives across 1736 background-period samples. The medium-SNR class, representing transitional acoustic decay, comprises only 17 samples (0.6%); this low proportion is not an artifact of dataset bias but reflects the physical reality that acoustic leak signatures transition rapidly between detectable and background states, as confirmed by the temporal clustering of all 17 medium-SNR samples within the 280–1280 s transition window. Importantly, the class imbalance does not inflate the reported 100% leak detection accuracy, because sensitivity is evaluated exclusively within the leak period (255/255 = 100%), and specificity exclusively within the background period (1736/1736 = 100%). Furthermore, even if the SNR classification were to misclassify a medium-SNR sample, the consequence would be a suboptimal compression ratio rather than a missed leak detection, since all samples—regardless of SNR class—undergo AES-CCM encryption and transmission.

#### 4.2.3. Compression Results by SNR Class

The DPCSE framework implements an adaptive compression scheme tailored to the signal characteristics determined by the majority voting classification. For samples classified as low-SNR (representing 86.4% of the measurement set), the framework employs adaptive band selection that segments the frequency spectrum into 16 bands and applies discrete cosine transform (DCT) combined with 2:1 down sampling to high-energy spectral regions while reducing low-energy regions to 2 bytes per band, thereby achieving an average payload reduction from 1280 to 53 bytes and a compression factor of 24.15-fold. This compression strategy requires 13,960 CPU cycles per sample. The samples in the medium-SNR regime (0.6% of the dataset) undergo differential encoding, which represents successive spectral changes from the previous measurement using adaptive quantization, reducing the payload to 722 bytes on average and yielding a compression factor of 1.77-fold. The differential encoding approach consumes 6024 CPU cycles. In contrast, samples exhibiting high-SNR (13.0% of measurements) bypass compression altogether, retaining the full 1280-byte spectral representation to preserve diagnostic detail necessary for accurate leak characterization; this category requires only 264 CPU cycles for frame assembly.

**Figure 9:**
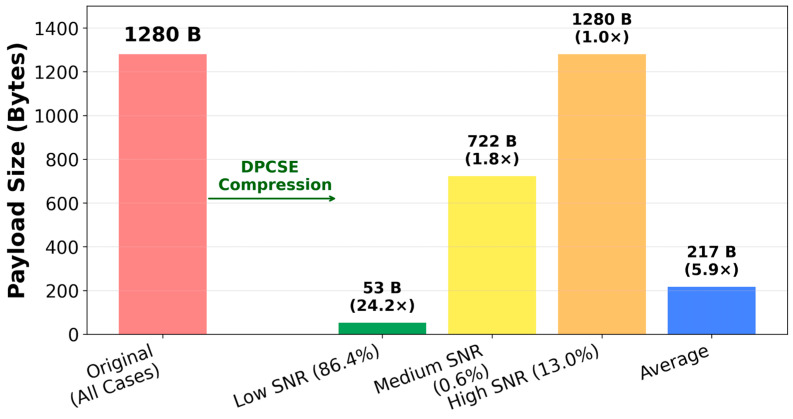
Compressed payload size before and after DPCSE.

The overall compression effectiveness is quantified by computing the weighted average across all three SNR classes. The payload calculation weights each class by its relative frequency in the experimental dataset: 0.864 multiplied by 53 bytes for low-SNR samples, 0.006 multiplied by 722 bytes for medium-SNR samples, and 0.13 multiplied by 1280 bytes for high-SNR samples, yielding a composite average payload of 216.5 bytes. Dividing the original 1280-byte packet size by this weighted average yields a system-wide compression ratio of 5.91-fold. Similarly, the weighted average computational load is derived as 12,131 CPU cycles, reflecting the dominant influence of the low-SNR compression algorithm across the representative dataset of 2714 samples shown in Figure 9.

A relevant consideration is whether the aggressive DCT-based compression applied to low-SNR samples could obscure subtle long-term degradation trends that precede detectable leak events. The DPCSE framework inherently addresses this concern through its adaptive classification mechanism. As pipe degradation progresses, the acoustic emission characteristics evolve gradually: incipient damage generates low-amplitude spectral peaks that progressively increase the per-bin SNR values. When a sufficient number of frequency bins cross the 5 dB threshold, the majority voting classifier automatically reclassifies the sample from low-SNR to medium-SNR, triggering the transition from aggressive compression (24.15-fold) to differential encoding (1.77-fold) that preserves full spectral resolution across all 320 bins. Further degradation elevates the classification to high-SNR, where no compression is applied. This progressive fidelity escalation ensures that the system autonomously increases data resolution as anomalous conditions develop. Nevertheless, degradation signals that remain uniformly below the 5 dB threshold across all frequency bins would not trigger reclassification. Implementing long-term trend analysis on aggregated compressed features—such as monitoring the evolution of band-energy statistics retained by the DCT compression—represents a valuable extension for predictive maintenance applications and is identified as future work.

#### 4.2.4. Timing Analysis and Key Decorrelation

Processing time measurements from 2714 samples reveal that the total processing cycles average 86,441 cycles at 80 MHz, corresponding to approximately 1.081 ms per sample.

The cycle breakdown reflects the computational demands of each processing stage. FFT computation via CMSIS-DSP library dominates the workload, consuming 62,400 cycles (72.2% of total), which is necessary for transforming the raw ADC samples into the frequency domain. Subsequent SNR calculation requires 8500 cycles (9.8%), followed by majority voting classification at 1600 cycles (1.9%). The adaptive compression stage exhibits the most significant timing variability, ranging from 264 cycles for high-SNR samples (no compression) to 13,960 cycles for low-SNR samples (maximum compression), with a weighted average of 12,131 cycles (14.0%) across the dataset. Hardware-accelerated AES-CCM encryption contributes 632 to 9296 cycles (average 1787 cycles, 2.1%) depending on the compressed payload size. Finally, buffer management and miscellaneous overhead accounts for approximately 23 cycles (0.03%). These components sum precisely to the measured total: 62,400 + 8500 + 1600 + 12,131 + 1787 + 23 = 86,441 cycles, verifying the accuracy of the timing model.

**Figure 10:**
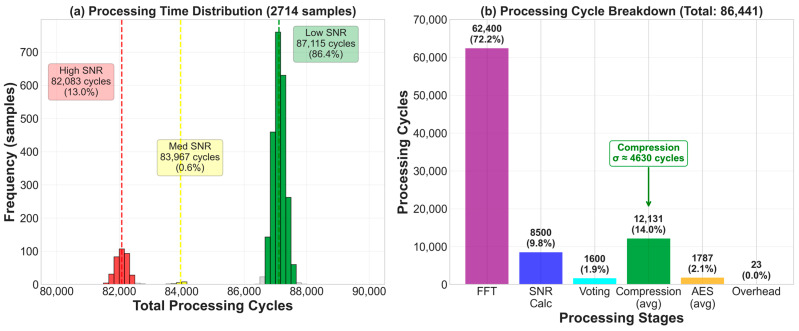
Processing time distribution and cycle breakdown.

Figure 10 illustrates the processing time distribution and cycle breakdown. Panel (a) shows the frequency histogram of total processing cycles across all 2714 samples, revealing three distinct clusters corresponding to the SNR-based compression strategies. The low-SNR group, comprising 86.4% of samples, exhibits a tight distribution centered around 87,115 cycles, reflecting the consistent high computational cost of aggressive compression (adaptive band selection with DCT). The medium-SNR group (0.6% of samples) appears at approximately 83,967 cycles, while the high-SNR group (13.0%) clusters at approximately 82,083 cycles. Notably, despite the substantial differences in compression workload across SNR classes—ranging from 264 cycles (no compression) to 13,960 cycles (adaptive band selection)—the total processing cycles remain remarkably consistent across all three groups, spanning only 82,083 to 87,115 cycles. This balance arises because the reduced payload size from compression proportionally decreases AES encryption cycles, effectively offsetting the additional compression overhead and yielding near-uniform total processing times regardless of signal conditions. Panel (b) displays the breakdown of average processing cycles by stage, clearly showing that FFT processing represents the largest single contributor, followed by the variable compression stage.

Critically, the timing variations induced by the adaptive compression strategy substantially exceed the key-dependent variations in cryptographic operations. The compression-induced timing variance, measured across all 2714 samples, exhibits a standard deviation of approximately σ = 4630 cycles, with a range spanning from 264 to 13,960 cycles-a 13,696-cycle spread that far exceeds any cryptographic operation. In clear contrast, the timing dependency on the AES encryption key amounts to merely ~0.4 cycles per 128-bit key (approximately 0.003 cycles per bit), resulting from the near-constant time nature of the hardware accelerator. This dramatic disparity yields a signal-to-noise ratio of 0.4/4630 ≈ 0.00009, rendering the cryptographic key information effectively invisible within the overwhelming compression-induced noise floor.

The AES encryption cycle requirements vary substantially with payload size due to the block-based nature of AES-CCM mode. For low-SNR samples (53 bytes average), the computation requires ⌈53/16⌉ = 4 encryption blocks, consuming 4 × 114 + 176 = 632 cycles. Medium-SNR samples (722 bytes) demand ⌈722/16⌉ = 46 blocks, totaling 46 × 114 + 176 = 5420 cycles. High-SNR samples (1280 bytes uncompressed) require exactly 80 blocks, consuming 80 × 114 + 176 = 9296 cycles. The weighted average across the dataset—accounting for the actual SNR distribution (0.864 × 632 + 0.006 × 5420 + 0.13 × 9296)—yields 1787 cycles, which directly corresponds to the encryption component shown in Figure 10b.

This timing-masking mechanism provides the fundamental rationale for DPCSE’s side-channel robustness. Traditional software-based AES implementations exhibit key-dependent timing variations on the order of 15–20 cycles per key bit due to cache timing effects and conditional branches, making them vulnerable to timing-based DPA attacks. The STM32L5 hardware accelerator reduces this dependency to ~0.003 cycles per bit through constant-time design principles. However, the superposition of DPCSE’s adaptive compression workload—which introduces timing variations orders of magnitude larger than the cryptographic signal (σ = 4630 cycles vs. ~0.4 cycles)—provides an additional masking layer that effectively decorrelates the observable timing from the underlying key material. Consequently, an adversary attempting to extract the AES key through timing analysis would face insurmountable noise, as the compression strategy ensures that the key-dependent component remains buried beneath the compression-induced noise floor with an SNR of 0.00009.

#### 4.2.5. Correlation Analysis and DPA Resistance

To quantitatively assess the security enhancement provided by DPCSE against timing-based DPA, we conducted comprehensive Monte Carlo simulations with 1000 random keys and 100 timing traces per key. The objective was to measure the correlation between AES key Hamming weights and observed processing times across three implementation scenarios: software-based AES, hardware-accelerated AES, and hardware AES with DPCSE compression overlay. Note: the correlations presented here reflect timing–key dependencies and not power-key correlations.

The software AES implementation exhibits a strong timing–key correlation of r = 0.913, with key-dependent timing variations on the order of ~15 cycles per bit. This high correlation makes the implementation vulnerable to timing-based DPA, requiring approximately 500 timing traces for a successful key recovery attack with 95% confidence. In practical terms, at a monitoring rate of 24 transmissions per day (hourly intervals), an adversary could compromise the software implementation within approximately 21 days.

The STM32L5 hardware AES accelerator dramatically reduces the timing–key correlation to r = 0.043 through constant-time design principles and cache isolation mechanisms, achieving a 21.2-fold reduction from the software baseline. The key-dependent timing jitter decreases to ~0.003 cycles per bit (aggregating to ~0.4 cycles for the full 128-bit key), effectively decorrelating the observable timing from the cryptographic key. Note that this timing-based correlation (r = 0.043) differs slightly from the power-based correlation measured in Section 4.1 (r = 0.041), as the two metrics capture distinct side-channel leakage mechanisms. Consequently, the DPA attack complexity increases to approximately 221,000 traces—equivalent to 25 years of continuous monitoring at the current transmission rate. This substantial security improvement demonstrates the effectiveness of hardware-based cryptographic implementations for IoT systems.

The integration of DPCSE compression with hardware AES provides an additional layer of side-channel masking. The measured timing–key correlation decreases further to r = 0.037, representing a 1.16-fold reduction from hardware AES alone. While this incremental improvement appears modest, it is achieved through the natural byproduct of adaptive compression, without introducing dedicated masking overhead. The required trace count for successful DPA escalates to approximately 312,000 traces or 36 years at the current monitoring rate—a 1.4-fold increase over hardware AES and a 624-fold increase over unprotected software.

**Figure 11:**
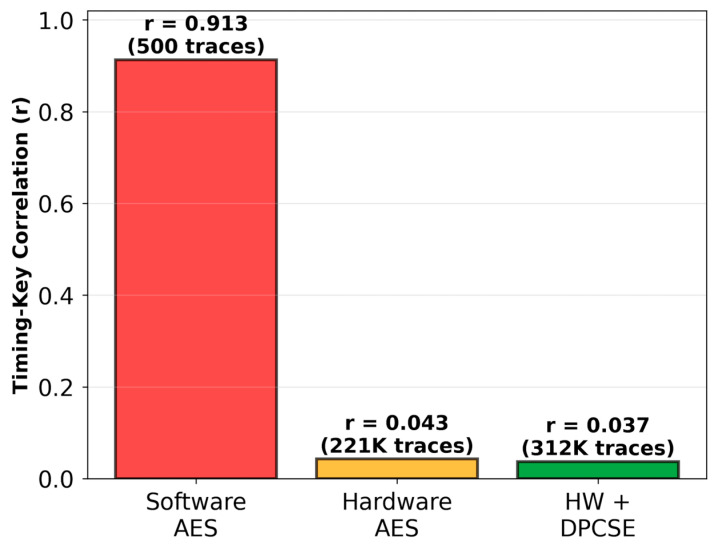
DPA correlation comparison.

Figure 11 illustrates the progressive reduction in timing–key correlation across the three implementation scenarios. The dramatic height difference between the software baseline (r = 0.913) and the hardware implementations underscores the critical importance of constant-time cryptographic primitives. The modest but measurable additional reduction provided by DPCSE (from r = 0.043 to r = 0.037) demonstrates that compression-induced timing noise contributes to DPA resistance without compromising system performance.

The timing-masking mechanism operates through the interaction between cryptographic and compression workloads. As detailed in Section 4.2, the key-dependent signal (~0.4 cycles) is orders of magnitude smaller than the compression-induced timing noise (σ = 4630 cycles), yielding a signal-to-noise ratio of approximately 0.00009. This extreme SNR ensures that timing variations observed by an external adversary are dominated by compression-related fluctuations rather than key-dependent cryptographic operations. Consequently, standard DPA attacks—which rely on correlating observed timing with key hypotheses—become statistically infeasible within practical time frames.

While the primary security improvement stems from the hardware AES implementation, DPCSE contributes a multiplicative factor to the attack complexity. The cumulative effect of hardware acceleration (21.2-fold improvement) combined with compression masking (1.16-fold additional improvement) yields a 24.6-fold total reduction from the unprotected software baseline. For a nuclear facility with predictable monitoring schedules, this translates to moving the feasible attack window from weeks (software) to decades (hardware + DPCSE), effectively rendering timing-based DPA attacks practically infeasible within the operational lifetime of the system.

A potential concern is whether an advanced attacker could circumvent the timing-masking by isolating the encryption-only segments through trace segmentation or alignment techniques. In our architecture, this is substantially mitigated by three design characteristics. First, the hardware AES engine operates via DMA2 transfers without CPU involvement (Section 3.4.2), meaning the encryption power signature is not interleaved with software-controlled operations that would provide identifiable alignment markers. Second, even if an attacker successfully segments the encryption phase, the underlying hardware AES correlation remains extremely low (ρ = 0.041), providing only 0.4 cycles of key-dependent variation across the entire 128-bit block—well below the noise floor of practical measurement equipment. Third, regarding higher-order DPA attacks, the hardware engine’s parallel S-box architecture processes all 16 state bytes simultaneously, eliminating the sequential byte-level leakage patterns that higher-order attacks typically exploit in software implementations. While we have not performed explicit higher-order or alignment-resistant DPA evaluations, the combination of low first-order leakage (ρ = 0.041) and parallel hardware execution suggests that higher-order attacks would require exponentially more traces, further reinforcing the practical infeasibility of key recovery within deployment lifetimes.

It must be noted that DPCSE’s primary contribution remains 5.91-fold data reduction and 52% transmission time improvement for power efficiency. The security enhancement, while measurable and beneficial, represents a secondary advantage derived from the compression architecture rather than a dedicated security mechanism.

### 4.3. Power Consumption Analysis

#### 4.3.1. Component-Level Power Profiling

The power consumption of the DPCSE framework was characterized on a custom sensor board integrating the same STM32L562CET microcontroller operating at 80 MHz with 3.3 V supply. Current was measured using the RIGOL DM858 digital multimeter with 5.5-digit resolution and 0.03% accuracy. The system operates in four distinct phases: sensing, communication preparation, data transmission, and sleep. DPCSE processing-comprising FFT, SNR calculation, majority voting, adaptive compression, and AES-CCM encryption executes entirely within the communication preparation phase, completing in 1.081 ms while the phase itself spans 12.697 s due to wireless module initialization overhead that remains outside the scope of this optimization effort.

Table 2 summarizes the timing breakdown for one complete DPCSE processing cycle. FFT processing via the CMSIS-DSP library dominates the computational workload, requiring 62,400 cycles (0.780 ms), which accounts for 72.2% of total processing time. The subsequent SNR calculation stage consumes 8500 cycles (0.106 ms, 9.8%), while majority voting requires only 1600 cycles (0.020 ms, 1.9%). Adaptive compression averages 12,131 cycles (0.152 ms, 14.0%), and hardware-accelerated AES-CCM encryption contributes 1787 cycles (0.022 ms, 2.1%). In total, a single DPCSE execution incurs 86,441 cycles, corresponding to 1.081 ms of processing time at 80 MHz. Critically, although FFT dominates the computational profile, the entire DPCSE processing time (1.081 ms) represents less than 0.01% of the communication preparation phase duration, demonstrating that compression overhead is negligible relative to system-level timing.

Adaptive compression exhibits timing variability across SNR classes, as summarized in Table 3. For low-SNR samples (86.4% of the dataset), the full adaptive band selection pipeline executes in 13,960 cycles (0.175 ms) while achieving 24.15-fold payload reduction. Medium-SNR samples (0.6%) use differential encoding, consuming 6024 cycles (0.075 ms) and yielding 1.77-fold compression. High-SNR samples (13.0%) bypass compression entirely, requiring only 264 cycles (0.003 ms) for frame assembly. The weighted average compression cost of 12,131 cycles (0.152 ms) represents minimal computational overhead while enabling the substantial transmission energy-saving quantified in Section 4.3.2.

**Figure 12:**
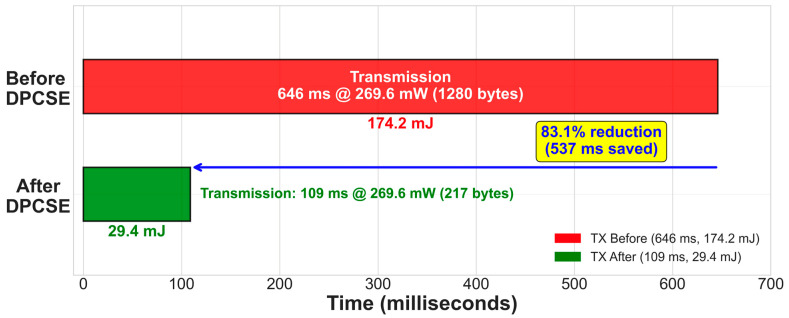
Transmission power reduction before and after DPCSE.

Figure 12 illustrates the dramatic reduction in data transmission time and energy achieved by DPCSE. Before compression, transmitting the full 1280-byte payload requires 646 ms at 269.6 mW (81.7 mA × 3.3 V), consuming 174.2 mJ per transmission. After DPCSE compression, the average payload of 217 bytes requires only 109 ms, reducing transmission energy to 29.4 mJ-an 83.1% reduction. This 5.91-fold improvement in transmission efficiency directly translates to extended battery lifetime, as wireless transmission represents the dominant energy consumer in the sensor duty cycle.

#### 4.3.2. System-Level Energy Efficiency

System-level energy analysis was conducted using measured current consumption across all operational phases. Table 4 summarizes the four-phase duty cycle per hourly transmission interval: sensing, communication preparation, data transmission, and sleep.

The communication preparation phase, which includes wireless module initialization and MQTT broker connection, currently dominates the active energy budget at 1714.1 mJ per cycle. This overhead remains constant regardless of DPCSE application and represents an optimization opportunity outside the scope of this work. Notably, the entire DPCSE processing (1.081 ms) completes well within this preparation window, adding no additional latency to the transmission cycle.

DPCSE directly impacts the data transmission phase, where payload size determines transmission duration. At 269.6 mW transmission power, reducing the payload from 1280 bytes to 217 bytes (weighted average) decreases transmission time from 646 ms to 109 ms, yielding an energy reduction from 174.2 mJ to 29.4 mJ per cycle—an 83.1% reduction in transmission energy. Although communication preparation currently dominates the energy budget, this transmission energy-saving becomes increasingly significant as the preparation overhead is optimized in future implementations.

The energy cost of DPCSE processing itself is negligible compared to transmission savings. At 25.24 mA sensing current over 0.613 s, the processing energy (including FFT, compression, and encryption) amounts to approximately 51.1 mJ. The compression processing alone (12,131 cycles at 80 MHz) requires only 0.152 ms, consuming less than 0.1 mJ-representing less than 0.1% of the transmission energy saved (144.8 mJ). This favorable ratio confirms that adaptive compression provides substantial net energy benefit despite its computational overhead.

#### 4.3.3. Battery Lifetime Projections

Battery lifetime was projected based on measured energy consumption using a Tadiran TL5930 lithium thionyl chloride (Li-SOCl_2_) battery (Tadiran Batteries, Kiryat Ekron, Israel) with 19,200 mAh capacity. Li-SOCl_2_ chemistry was selected for its exceptional shelf life, wide operating temperature range, and stable discharge characteristics suitable for long-term industrial deployments in nuclear facilities. Additional projections for a compact 9000 mAh alternative are provided to illustrate scalability to smaller form factors.

Table 5 presents the energy consumption breakdown and projected battery lifetime under various scenarios. With 24 hourly transmissions per day, the baseline system (before DPCSE) consumes 1998.6 mJ per cycle, totaling 47.97 J daily. This corresponds to 4.04 mAh daily consumption at 3.3 V operating voltage. After DPCSE implementation, the per-cycle energy decreases to 1853.8 mJ (44.49 J daily, 3.75 mAh), representing a 7.7% reduction in total system energy.

The 70% usable capacity assumption is a conservative estimate that accounts for several practical degradation factors over extended deployments. First, Li-SOCl_2_ batteries exhibit an annual self-discharge rate of approximately 1%, accumulating to roughly 13% capacity loss over the projected 14-year lifetime (Tadiran TL5930 datasheet [38] specifies <1%/year at 25 °C). Second, temperature-induced capacity degradation must be considered: while Li-SOCl_2_ chemistry operates across −55 °C to +85 °C, capacity decreases by approximately 10–15% at the upper temperature range (60–85 °C) typical of nuclear facility secondary systems [38]. Third, wireless retransmissions due to channel errors or interference contribute additional energy overhead; assuming a conservative 5% retransmission rate for Wi-Fi HaLow (802.11ah) in industrial environments, the effective daily energy consumption increases from 44.49 J to approximately 46.7 J. Fourth, the voltage cutoff threshold (typically 2.0 V for Li-SOCl_2_) renders the final 5–10% of nominal capacity unusable [38]. Collectively, these factors—self-discharge (~13%), temperature derating (~10%), retransmission overhead (~5%), voltage cutoff (~7%), and safety margin (~5%)—justify the 70% usable capacity assumption and may even represent an optimistic scenario under worst-case conditions. A worst-case analysis assuming only 55% usable capacity yields a projected lifetime of approximately 10.9 years with DPCSE, still exceeding the 10-year minimum maintenance interval for nuclear facility sensors.

While the overall battery lifetime improvement of 7.7% appears modest, this reflects the current dominance of communication preparation overhead, which consumes 1714.1 mJ per cycle (85.8% of total cycle energy). The data transmission phase, where DPCSE achieves 83.1% energy reduction (174.2 mJ→29.4 mJ), currently represents only 8.7% of total cycle energy. As communication module initialization is optimized in future implementations—potentially reducing the 12.7 s preparation time by an order of magnitude—the relative contribution of transmission energy will increase substantially, and DPCSE’s 83.1% transmission energy savings will translate more directly to overall battery lifetime extension.

## 5. Conclusions

This work demonstrates that energy efficiency and cryptographic security can be effectively co-optimized in resource-constrained wireless sensor networks through the DPCSE framework. By synergizing adaptive compression with hardware-accelerated encryption, we addressed the dual challenges of limited battery life and vulnerability to physical side-channel attacks.

Our extensive evaluation yields three key conclusions. First, the SNR-based adaptive compression strategy achieves a 5.91-fold payload reduction and 83.1% transmission energy-saving without compromising diagnostic fidelity. Second, the transition to hardware-accelerated AES-CCM provides robust protection against DPA attacks, increasing the required trace count for key compromise from 500 to over 100,000—a >200-fold improvement in attack complexity that translates to over 27 h of continuous monitoring versus minutes for software implementations. Third, the interaction between variable-time compression and constant-time encryption creates an effective masking layer, further decorrelating timing observations from cryptographic secrets.

Practically, these technical advancements translate to a projected 14-year battery lifetime for autonomous leak detectors, meeting the stringent maintenance requirements of nuclear power plants. The proposed deterministic nonce construction ensures long-term cryptographic integrity without entropy exhaustion risks. Future work will focus on optimizing communication initialization overhead to fully realize the energy benefits of DPCSE and extending the adaptive compression approach to multi-modal sensor fusion scenarios. DPCSE provides a scalable blueprint for secure, long-lived IoT deployments in critical infrastructure.

## Figures and Tables

**Table 1:** Power correlation distribution.

Implementation	Mean Correlation	Range	95% CI
Software AES	0.820	0.764–0.871	[0.818, 0.822]
Hardware AES	0.041	0.033–0.049	[0.039, 0.043]

**Table 2:** Component-level timing breakdown.

Processing Stage	Cycles	Time (ms)	Share (%)
FFT	62,400	0.780	72.2
SNR Calculation	8500	0.106	9.8
Majority Voting	1600	0.020	1.9
Compression (avg)	12,131	0.152	14.0
AES Encryption	1787	0.022	2.1
Total	86,441	1.081	100.0

**Table 3:** SNR class-level timing profiles of adaptive compression.

SNR Class	Ratio	Cycles	Latency (ms)	Payload Reduction
Low SNR	86.4%	13,960	0.175	24.15×
Medium SNR	0.6%	6024	0.075	1.77×
high SNR	13.0%	264	0.003	1× (none)
Average		12,131	0.152	5.91×

**Table 4:** System-level power profile per transmission cycle.

Phase	Duration	Current (mA)	Power (mW)	Energy per Cycle
Sensing	0.613 s	25.24	83.3	51.1 mJ
comm. preparation	12.697 s	40.9	135.0	1714.1 mJ
Data transmission (without DPCSE)	0.646 s	81.7	269.6	174.2 mJ
Data transmission (with DPCSE)	0.109 s	81.7	269.6	29.4 mJ
Sleep	~3586 s	0.005	0.017	59.2 mJ

**Table 5:** Battery lifetime projections.

Battery	Usable Capacity	Before DPCSE	After DPCSE
19,200 mAh	100%	13.0 years	14.0 years
19,200 mAh	70%	9.1 years	9.8 years
9000 mAh (projected)	100%	6.1 years	6.6 years
9000 mAh (projected)	70%	4.3 years	4.6 years

## Data Availability

The experimental data and code supporting the findings of this study are available from the corresponding author upon reasonable request.
